# Editorial: New Roles of Autophagy Pathways in Cancer

**DOI:** 10.3389/fonc.2021.726989

**Published:** 2021-07-07

**Authors:** Waleska K. Martins, Claudio M. Fader, Eugenia Morselli, Daniel Grasso

**Affiliations:** ^1^ Laboratory of Cell and Membrane, Anhanguera University of Sao Paulo, São Paulo, Brazil; ^2^ Laboratorio de Biología Celular y Molecular, Instituto de Histología y Embriología (IHEM), Universidad Nacional de Cuyo, CONICET, Mendoza, Argentina; ^3^ Facultad de Odontología, Universidad Nacional de Cuyo, Mendoza, Argentina; ^4^ Laboratory of Autophagy and Metabolism, Department of Physiology, Faculty of Biological Sciences, Pontificia Universidad Católica De Chile, Santiago, Chile; ^5^ Autophagy Research Center, Santiago de Chile, Chile; ^6^ Instituto de Estudios de la Inmunidad Humoral (IDEHU), CONICET, Universidad de Buenos Aires, Buenos Aires, Argentina; ^7^ Departamento de Ciencias Biológicas, Facultad de Farmacia y Bioquímica, Universidad de Buenos Aires, Buenos Aires, Argentina

**Keywords:** autophagy, cancer, research topic, molecular pathways, age-related disease

From a simplistic point of view, autophagy is a self-degradative process that relies on lysosomes for the removal of cytoplasmic bulk cargo and damaged organelles, such as mitochondria. Further on its homeostatic role, autophagy acts as a catabolic process that promotes cellular resilience in conditions of nutrient deprivation and energy depletion. A body of literature has established a crucial role of autophagy in a whole plethora of different physiological processes ranging from homeostasis maintenance, development, and differentiation, among others. In the last two decades, the complexity of autophagy regulation has grown exponentially. Indeed, the literature recognizes canonical and non-canonical autophagic pathways that lead to the degradation and clearance of non-specific or specific cargos (selective autophagy) depending on the cellular context. Due to the fundamental role of autophagy in homeostasis maintenance, it is not surprising its recognized etiologic role in age-related diseases, including cancer. In cancer, autophagy has a dual function, acting as a cell survival mechanism (e.g. favoring the growth of established tumors) or as a tumor suppressor (e.g. preventing the accumulation of damaged proteins and organelles). Thus, the relationship of autophagy with carcinogenesis is complex and, in most cases, it is considered a context-dependent process.

This collection compiles some of the most recent advances in the knowledge of the autophagic pathway and its involvement in human cancer development. Carcinogenesis implies proliferation, tissue invasion, vascularization, and modulation of the immune system. Herein, we expanded our knowledge about autophagy in carcinogenesis, showing how it has been engaged in various processes, including tumor progression, cancer-related thrombosis and metastasis, cancer dormancy linked to stem cell behavior and quiescence, epithelial-to-mesenchymal transition (EMT), intercellular communications, cell-stroma interactions, and tumor microenvironment (TME), immune responses, treatment resistance and tumor-adaptative response.

Interestingly, proliferation, invasion, vascularization, and immune modulation, which are characteristics of cancer, are also present in trophoblast cells during placentation. However, while these processes are strictly regulated during placentation, in the context of tumor growth the regulation is lost. Carvajal et al. discuss similarities and differences between carcinogenesis and placentation and the role of autophagy in the processes. As previously mentioned, autophagy can be a bulk process, or highly selective and Cerda-Troncoso et al. review the pro-tumorigenic roles of the better-described autophagy receptors such as p62/SQSTM1, NBR1, NDP52, and OPTN, which are overexpressed in cancer, and could be considered as new therapeutic targets against tumor growth. Additionally, Xie et al. describe the regulation of the autophagy-mediated selective turnover of mitochondria (mitophagy) in the highly mortal pancreatic ductal adenocarcinoma. Mitochondria quality control is critical for cell homeostasis and authors show the dual roles that the different mitophagy pathways (e.g., PINK-PRKN, BNIP3L/NIX, FUNDL1, and BNIP3) have in carcinogenesis and treatment of pancreatic cancer. Consistently, considering the different types of autophagy, Rios et al. analyze the contribution of chaperone-mediated autophagy in carcinogenesis. In an exciting emerging topic, Hernández-Cáceres et al. explore the involvement of cell mechanics during oncogenesis in autophagy regulation and its implication in disease progression. In response to the complex mechano-environment of tumor burden, cells activate mechanosensitive protein complexes and related-active cytoskeleton processes that impact the autophagy machinery and disease progression. On the other hand, instead of mechanical stimuli, autophagy can be regulated by external organisms such as viruses. Suares et al. and Ducasa et al. show how oncoviruses modulate the autophagy machinery enhancing viral survival and replication that eventually culminates in cell transformation. Altogether, these works suggest that a deeper and detailed comprehension of autophagy mechanisms might pave the way to explore precision therapy approaches against specific tumors.

Cancer is a tissular disease where cancerous cells are in dynamic communication with the different actors of TME and this relationship ultimately determines most of the tumor behavior. Consequently, many researchers focus their work to uncover the complexity of TME and its relationship with cancer cells. Coelho et al. describe the role of WNT signaling, in response to TME stress, in the regulation of the dichotomic fate between EMT and autophagy in glioblastoma. The autophagy pathway is also key for the TME establishment in oral squamous cell carcinoma, as described in Peña-Oyarzún et al. Furthermore, despite its actions over tumor cells, autophagy is also relevant for the communication among tumoral cells, TME, and the whole body. This autophagic role, as a means for intercellular communication, is highlighted in Bustos et al. where they describe the non-autophagic functions of the pathway, focusing on the new field of the autophagy-dependent secretion and its important implication for cancer fate (including the TME), the immune response and the biogenesis and secretion of extracellular vesicles (EVs). There seems to exist a strong relationship between the autophagy machinery and EVs, especially the exosomes that are EVs of endosomal origin. The state of the art concerning the autophagy-EVs complex associated with TME was investigated in Colletti et al. and Papademetrio et al. Noteworthy, the tumor-associated immune system is also reached by the plethora of different EVs of TME as it is shown in Colletti et al. and Papademetrio et al. Independently of the EVs, autophagy plays a key role in the tumor-associated immunology system, according to de Souza et al. The vast literature compiled in de Souza et al. reveals the key role of autophagy in tumor immunogenicity and how it engages TME, which might provide new insights into mitigating tumor relapse.

Beyond its pro-tumor role in carcinogenesis, autophagy highlights also as a promisor target for diagnosis and prognosis of cancer as discussed in this Research Topic. Meng et al., Lyu et al. and Deng et al. describe different autophagy-related genetic signatures as potential prognostic tools for neuroblastoma, bladder urothelial carcinoma, and pancreatic cancer, respectively. On the dark side, Akkoc et al. discussed autophagy’s role in cancer dormancy, which eventually contributes to metastasis and relapse, the leading cause of cancer-related deaths. Thrombotic events due to an enhanced thrombotic state are a severe complication among patients suffering from different types of cancers. Interestingly, Hill et al. discuss autophagy´s role along with all the coagulation components system and highlights the related implications regarding the TME and tumor development. Moreover, it is well accepted that autophagy may elicit tumor therapeutic resistance, and thus, it has been considered as a pharmacologic target to alleviate tumor relapse. Towards this end, Lai et al. described how metformin might overcome the autophagy-mediated resistance to Sorafenib treatment in hepatocellular carcinoma patients. On the other hand, Xiao et al. discussed how to abrogate cytoprotective autophagy (i.e., by genetic and pharmacologic means) to improve antitumor therapeutic approaches. Similarly, Jandrey et al., based on compiling articles, contributed to deciphering the cancer resilience of glioblastoma cells to conventional chemotherapies in terms of the pro-survival autophagy proficiency. Furthermore, Martins et al. reveal how photodynamic therapy (PDT) mediated-oxidative stress may induce autophagy in tumor cells, and how lysosomal photodamage might trigger autophagy as a regulated cell death mechanism, improving the clinical outcome of PDT-treated patients.

Carcinogenesis is an age-related process, and as humans extend their life expectancy, the incidence of cancer will continue to increase. Although lots of progress has been made in the diagnosis and treatment of many human cancers, we are still far from a panacea. Thus, morbidity and mortality related to cancer remain a global issue. The milestone contributions presented in this Research Topic regarding the “new roles of the autophagy pathway in cancer” reveal the great advancement made in the underlying molecular mechanism of autophagy and its implication for the comprehension of cancer. Most works unveil an intricate and complex role of autophagy in tumoral cells, TME, and even the communications among them. Altogether, this knowledge provides us a glimpse of new therapeutic options to be explored, mainly in cancer resilience. Furthermore, since autophagy in cancer is known to be a double edge sword, it is of great importance to get a deep insight of its role during carcinogenesis, to design specific therapeutic approaches. Finally, it is an exciting time of great discoveries about the autophagic pathway, and more importantly, those discoveries bring hope and assure great benefits in the long fight against a devastating disease like cancer.

## Author Contributions

WM, CF, EM, and DG contributed equally to this Research Topic by acting as guest editors, with professionalism, sharing their thoughts and experience, working in a group, and writing the editorial. All authors contributed to the article and approved the submitted version.

## Funding

DG receives financial support from University of Buenos Aires (UBACyT 2020—20020190200047BA) and the *Agencia Nacional de Promoción Científica y Técnica* (ANPCyT—PICT-2018-02220).

## Conflict of Interest

The authors declare that the research was conducted in the absence of any commercial or financial relationships that could be construed as a potential conflict of interest.

